# Metabolic Role of GABA in the Secretory Function of Pancreatic β-Cells: Its Hypothetical Implication in β-Cell Degradation in Type 2 Diabetes

**DOI:** 10.3390/metabo13060697

**Published:** 2023-05-27

**Authors:** Jorge Tamarit-Rodriguez

**Affiliations:** Biohemistry Department, Medical School, Complutense University, 28040 Madrid, Spain; tamarit@ucm.es

**Keywords:** GABA, GABA metabolism, GABA and stimulus secretion-coupling of glucose-induced insulin secretion, β-cells, pancreatic islets, insulin secretagogues, branched-chain alpha-ketoacids a, alpha-amino acids, insulin secretion

## Abstract

The stimulus-secretion coupling of a glucose-induced release is generally attributed to the metabolism of the hexose in the β-cells in the glycolytic pathway and the citric acid cycle. Glucose metabolism generates an increased cytosolic concentration of ATP and of the ATP/ADP ratio that closes the ATP-dependent K^+^-channel at the plasma membrane. The resultant depolarization of the β-cells opens voltage-dependent Ca^2+^-channels at the plasma membrane that triggers the exocytosis of insulin secretory granules. The secretory response is biphasic with a first and transient peak followed by a sustained phase. The first phase is reproduced by a depolarization of the β-cells with high extracellular KCl maintaining the KATP-channels open with diazoxide (triggering phase); the sustained phase (amplifying phase) depends on the participation of metabolic signals that remain to be determined. Our group has been investigating for several years the participation of the β-cell GABA metabolism in the stimulation of insulin secretion by three different secretagogues (glucose, a mixture of L-leucine plus L-glutamine, and some branched chain alpha-ketoacids, BCKAs). They stimulate a biphasic secretion of insulin accompanied by a strong suppression of the intracellular islet content of gamma-aminobutyric acid (GABA). As the islet GABA release simultaneously decreased, it was concluded that this resulted from an increased GABA shunt metabolism. The entrance of GABA into the shunt is catalyzed by GABA transaminase (GABAT) that transfers an amino group between GABA and alpha-ketoglutarate, resulting in succinic acid semialdehyde (SSA) and L-glutamate. SSA is oxidized to succinic acid that is further oxidized in the citric acid cycle. Inhibitors of GABAT (gamma-vinyl GABA, gabaculine) or glutamic acid decarboxylating activity (GAD), allylglycine, partially suppress the secretory response as well as GABA metabolism and islet ATP content and the ATP/ADP ratio. It is concluded that the GABA shunt metabolism contributes together with the own metabolism of metabolic secretagogues to increase islet mitochondrial oxidative phosphorylation. These experimental findings emphasize that the GABA shunt metabolism is a previously unrecognized anaplerotic mitochondrial pathway feeding the citric acid cycle with a β-cell endogenous substrate. It is therefore a postulated alternative to the proposed mitochondrial cataplerotic pathway(s) responsible for the amplification phase of insulin secretion. It is concluded the new postulated alternative suggests a possible new mechanism of β-cell degradation in type 2 (perhaps also in type 1) diabetes.

## 1. Metabolic Role of GABA in the Mechanism of Stimulation of Insulin Secretion

### 1.1. Introduction

The stimulus-secretion coupling of the glucose-induced release of gamma-aminobutyric acid (GABA) is generally attributed to the metabolism of hexose in pancreatic β-cells in the glycolytic pathway and the citric acid cycle. Glucose metabolism generates an increased cytosolic concentration of ATP and ATP/ADP ratio that closes ATP-dependent K^+^-channels at the plasma membrane via the interaction of ATP with the regulatory Kir6.2 channel subunit [1]. The resultant depolarization of β-cells opens voltage-dependent Ca^2+^-channels in the plasma membrane, which leads to an increase in cytosolic cation concentration and triggers the exocytosis of insulin secretory granules [1]. This secretory response evolves in time as a biphasic secretion with a first and transient peak of an approximately 10 min duration (in rat perifused islets), followed by a sustained phase lasting as long as the stimulus. Whereas the first transient peak can be reproduced by a simple depolarization of the β-cells with high extracellular KCl maintaining the opening of K_ATP_ channels with diazoxide (triggering phase) [2], the sustained phase depends on the participation of metabolic signals that remain to be determined (amplifying phase) [3,4]. Given that extracellular ATP alone (10 mM) triggers a biphasic insulin secretion in KCl-permeabilized islets [5], the metabolic signal responsible for the sustained phase may involve any enzymatic pathway that is capable of triggering ATP synthesis. Our group has been investigating for several years the participation of β-cells in GABA metabolism, together with the mechanisms of glucose (a β-cell-specific nutrient secretagogue) and other “metabolic” secretagogues (physiological metabolites at supraphysiological concentrations) in the simulation of insulin secretion.

γ-aminobutyric acid (GABA) is exclusively abundant in the β-cells of pancreatic islets, which are equipped with the enzymes required to synthesize and metabolize GABA in the GABA shunt [6], including (1) glutamic acid decarboxylase isoenzymes (cytosolic GAD65 and GAD67) which generate GABA via L-glutamate decarboxylation; (2) GABA transaminase (GABAT), a mitochondrial enzyme exchanging an amino group between GABA and α-ketoglutarate (αKG or 2-oxoglutarate, 2-OG) to give succinic acid semialdehyde (SSA) and L-glutamate; (3) NAD^+^-dependent SSA dehydrogenase that oxidizes SSA to succinic acid, which is further metabolized in the Krebs cycle; and (4) NADPH-dependent SSA reductase that reduces SSA to γ-hydroxybutyric acid and competes with the previous enzyme for the same substrate.

**Figure 1:**
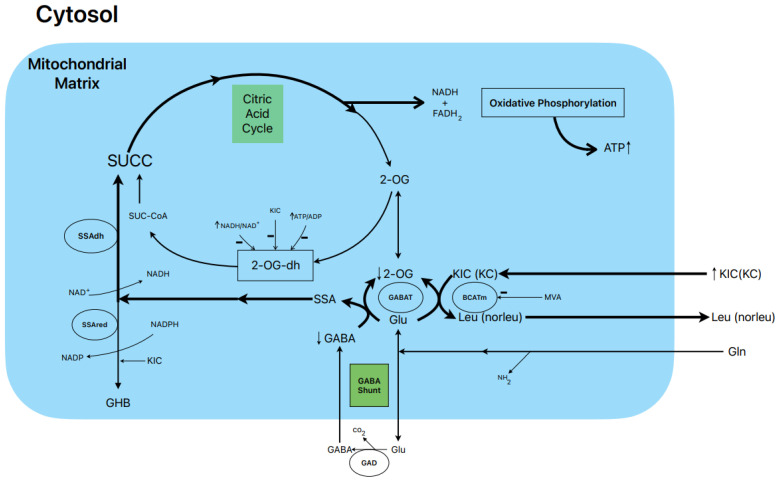
Postulated metabolic pathway leading to the stimulation of BCKA-induced insulin secretion.

**Figure 2:**
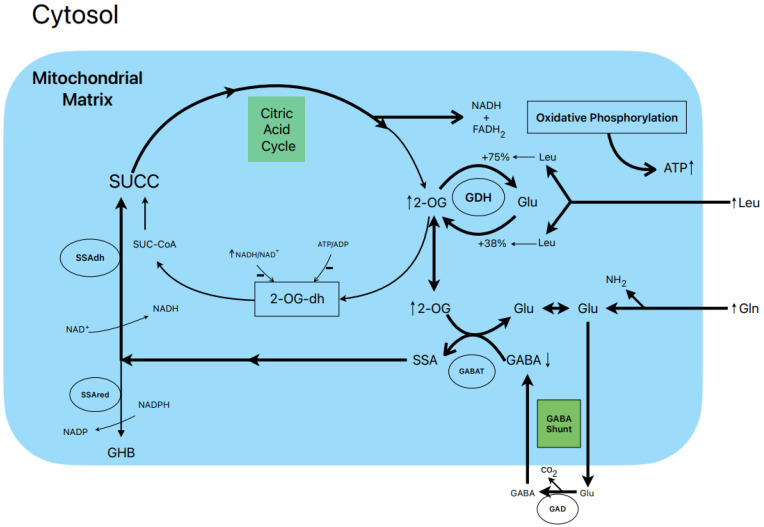
Postulated metabolic pathway leading to the stimulation of insulin secretion by L-leucine plus L-glutamine.

GABA’s functional role in β-cells has been discussed for many years [7], but there is no conceptual consensus on its participation in the mechanism of biphasic insulin secretion triggered by glucose and other known metabolic stimuli (fundamentally L-leucine + L-glutamine and branched-chain 2-oxoacids (BCKAs)). So far, the most accepted hypothesis seems to be autocrine regulation via the release (or co-secretion with insulin) of GABA through the stimulation of either GABA-A or GABA-B receptors [8,9] (See Figure 1 and Figure 2 of reference [7] for a schematic and immunostaining images of GAD65 and GABA in islets and B-cells). Additionally, a possible mechanism involving paracrine regulation of glucagon secretion by GABA, which is co-secreted with insulin, through the activation of GABA_A_ receptors of α-cells has been postulated [10]. However, in this paper, we limit our review to experimental data on the participation of intracellular GABA in islet metabolism.

### 1.2. Effect of Glucose Stimulation of Insulin Secretion on Islet GABA Content

Since the pioneering work of M. Erecinska [11] suggesting an energetic role for GABA in β-cells contributing to the maintenance of islet ATP/ADP ratio, some progress has been achieved. A first important finding is the demonstration that islet GABA synthesis in vitro is dependent on the extracellular concentration of L-glutamine [12]: it was found to reach a maximum between 0.5 to 1 mM, which is the physiological value of plasma L-glutamine concentrations [13]. This means that rat islet GABA content is regularly maintained at relatively high values, together with L-glutamate and L-aspartate, in comparison with other L-amino acids [13]. A detailed study on the effect of 20 mM glucose on the spectrum of islet plasma L-amino acids showed strong, specific, and significant decreases in islet GABA content with respect to the values at 0 mM glucose in the range of L-glutamine concentrations used (0.0, 0.5, and 10.0 mM). The simultaneous release of GABA at either 0.5 or 10.0 mM L-glutamine was also strongly reduced [14]. It was concluded that the suppression of islet GABA content did not depend on an increased rate of release, but it was produced by an increase in its metabolic rate. This was supported by the finding that inhibition of the glucose metabolism by D-mannoheptulose blocked glucose’s capacity to suppress islet GABA content (Figure S1 in [14,15]). A significantly positive, linear correlation was found between islet content and GABA release for the values obtained at both 0 and 20 mM glucose: the averaged GABA release at 0 mM glucose was higher than at 20 mM glucose at 0.5 and 10.0 mM L-glutamine. This suggests that the lower the GABA content, the lower the rate of release [12,14]. The linear correlation obtained between GABA content and release has been confirmed by other authors who supplied experimental evidence of the mediation of GABA release and uptake by the membrane anion transporters, VRAC and TauT [8]. As in previous studies, our data speak against the co-secretion of insulin and GABA under glucose stimulation. By contrast, our data do not predict an increase in GABA release by high glucose, but a decrease [14]. Notwithstanding, the remaining release of GABA through the postulated anion transporters might contribute to the pulsatility of the temporarily stimulated insulin secretion [8].

### 1.3. Effects of Branched-Chain 2-Oxoacid (BCKA) Stimulation of Insulin Release on Islet GABA Content

Besides glucose, branched-chain 2-oxoacids (α-ketoisocaproic acid or KIC and -α-ketocaproic acid or KC, which are the deaminated products of L-leucine and L-norleucine, respectively, and other branched-chain α-keto acids) are potent “metabolic” stimuli of islet insulin secretion at supraphysiological concentrations in the absence of glucose. They induce a biphasic secretion of insulin similar to that of glucose, but of variable magnitude and, similar to glucose, strongly decrease the islet content of GABA [16]. In a previous study, the branched-chain 2-oxoacids (KIC and KC) were aminated back to their corresponding amino acids (L-leu and L-norLeu, respectively), which diffused and accumulated in the incubation medium without increasing their islet content [16]. These results suggested that the amination of these branched-chain 2-oxo acids was coupled to the deamination of L-glutamate to αKG by α-branched-chain amino acid transaminases (BCATs: cytosolic or mitochondrial). This hypothesis was supported by the suppression of BCKA-stimulated insulin secretion with generic or more specific inhibitors of BCKATs [17]. Moreover, a recent publication demonstrated that a general knockout of BCKATm (mitochondrial isoform) in mice blocked insulin secretion stimulation in the majority of regularly used BCKAs without affecting glucose stimulation [18]. These results further supported the contention of the important role of BCKA amination in the stimulation of insulin secretion. However, the authors did not investigate the effects of BCKAs on islet GABA metabolism.

### 1.4. Effects of L-Leucine Plus L-Glutamine Stimulation of Insulin Release on Islet GABA Content

Another example of a long-known, strong “metabolic” secretagogue mixture is the combination of L-glutamine plus L-leucine, with the latter at supraphysiological concentrations. L-leucine has been shown to be an allosteric activator of mitochondrial L-glutamate dehydrogenase (GDH) catalyzing the conversion between L-glutamate and α-KG [19]. In previous research, L-leucine alone (10 mM) stimulated a predominantly first phase of insulin secretion in comparison with glucose (20 mM), but in combination with 10 mM glutamine, it triggered a stronger biphasic secretion [13,20]. L-leucine (10 mM) did not modify islet GABA content in the absence of extracellular L-glutamine but significantly stimulated the islet concentrations of L-glutamate and L-aspartate. At 5 and 10 mM glutamine, the islet content of all recorded amino acids increased several folds, and that of GABA was significantly decreased by L-leucine (−38%), 10 mM BCH (GDH activator) (−56%), and 10 mM allylglycine (GAD inhibitor) (−42%) [13].

### 1.5. Conclusions and Prospects

In these three main examples of nutrient and metabolic secretagogues, their metabolism shares a common metabolic step: the diversion of αKG to the GABA shunt where it will first be transaminated with GABA by GABAT to render L-glutamate and semialdehyde succinic acid (SSA). SSA will then be oxidized by semialdehyde succinic acid dehydrogenase (SSAdh) to succinic acid, which will enter the Krebs cycle for further oxidation. The resulting ATP synthesis leads to an elevated ATP/ADP ratio and the closure of K^+^_ATP_ channels that initiate the stimulation of insulin secretion.

A confirmation of these hypothetical mechanisms presupposes that interference with the GABA shunt may alter insulin responses to nutrient and metabolic secretagogues, as well as their capacity to increase their mitochondrial production of ATP. For that purpose, gabaculine and allylglycine have been used as GABAT and GAD65 inhibitors, respectively, to check their effects on insulin secretion, adenine nucleotide concentrations, and GABA metabolism.

### 1.6. Postulated Metabolic Pathway Leading to the Stimulation of BCKA-Induced Insulin Secretion (Figure 1)

In our study, 10 mM KIC induced biphasic insulin secretion in the absence of glucose, and its magnitude was not modified by either 0.5 or 10.0 mM L-glutamine. Gabaculine (0.25 mM) diminished the height of the first peak of secretion and significantly suppressed the second phase of sustained release by 33% [16]. KIC increased ^14^CO_2_ production by 27% and 66% in the presence of 0.5 and 10.0 mM L-(U-^14^C) glutamine, respectively [16]. Considering that the rate of L-(U-^14^C) glutamine oxidation is stoichiometric with the amount of GABA synthesized [13], this would facilitate the replenishment of the intracellular pool of GABA. On the other hand, 0.25 mM gabaculine did not modify the rate of L-glutamine oxidation in the presence of KIC, whereas 40 µM gabaculine strongly suppressed GABAT activity in rat islet homogenates [16]. Gabaculine (0.25 mM) also blocked, within 59%, the abrupt decrease in intra-islet oxygen tension caused by 10 mM KIC, reflecting that the contribution to islet oxygen consumption promoted by α-keto acid is partially attributable to an increased flux in the GABA shunt and part of the Krebs cycle [16].

### 1.7. Postulated Metabolic Pathway Leading to the Stimulation of Insulin Secretion by L-Leucine plus L-Glutamine (Figure 2)

L-leucine (10 mM) alone does not stimulate the oxidation of ^14^CO_2_ production in the presence of 0.5 and 10.0 mM L-(U-^14^C) glutamine [13]. However, other authors have shown that BCH (GDH activator) and some amino acids (L-isoleucine and L-norvaline, at 20 mM) stimulated ^14^CO_2_ production from islets that were pre-labelled with 1 mM L-(U-^14^C) glutamine [19]. Nevertheless, 10 mM L-leucine strongly suppressed islet GABA content in the presence of 0.5 and 10.0 mM L-glutamine [13]. The L-glutamine potentiation of L-leucine stimulated insulin secretion is generally assumed to be due to the production of αKG secondary to GDH stimulation. According to our own data, the equilibrium of GDH activity in islet homogenates favors the amination (A) reaction over the deamination (D) reaction (A/D = 8.2) [13]. L-leucine (10 mM) increases the ratio more in favor of the amination reaction (A/D = 10.1; *p* < 0.05 vs. in the absence of Leu) [13]. This condition would not facilitate an optimal αKG concentration to increase the net flux through GABAT. Therefore, it is possible that GABAT requires a higher supply of GABA (higher medium L-glutamine concentration) than KIC. In fact, KIC induces a strong decrease in cellular GABA in the absence of extracellular L-glutamine, whereas L-leucine fails to do so [13]. Moreover, the dimethyl ester of α-ketoglutarate (dmKG), a membrane-permeable analogue of αKG, was shown to increase the islet GABA content at 5 mM but decreased it significantly at higher concentrations in the absence or presence of L-leucine [13].

### 1.8. Postulated Metabolic Pathway Leading to the Stimulation of Insulin Secretion by Glucose (Figure 3)

**Figure 3:**
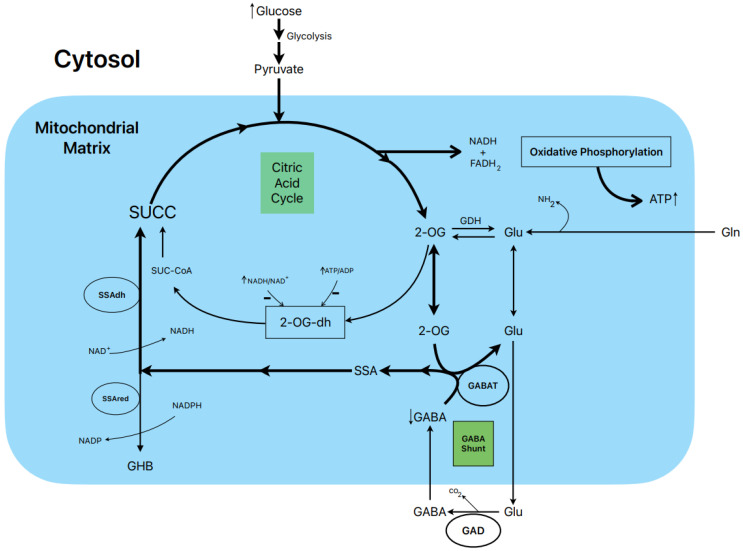
Postulated metabolic pathway leading to the stimulation of insulin secretion by glucose.

In rat islets, the biphasic stimulation of insulin secretion by 20 mM glucose was partially suppressed by 1 mM gabaculine in the presence of 10 mM L-glutamine, and it was also decreased by 20 mM allylglycine (reference [14], Figure 3C and Figure 2S, respectively). These experimental data support that the metabolic flux in the GABA shunt also contributes to the stimulated secretion. This is also supported by the reduction in islet ATP content and ATP/ADP ratio at 20 mM glucose induced by 1 mM gabaculine in the absence and presence of 1 and 10 mM L-glutamine [14].

An important argument to support what might be named the “GABA metabolic hypothesis” for the stimulation of insulin secretion by glucose and other non-physiological secretagogues is why the GABA shunt might be required to participate in the metabolic stimulation of insulin secretion. We have proposed that, at least in islet β-cells, the flux in the Krebs cycle is limited by the lower expression of the 2-oxo-glutarate (αKG) dehydrogenase gene compared with the gene of its competitor enzyme, GABAT, in the GABA shunt for their common substrate 2-oxo-glutarate (αKG) (see supplementary Figure S4 in reference [14]). In a model of “KCl-permeabilized islets” [5], we demonstrated that 5 mM αKG stimulated a sustained phase of insulin secretion after the peak of release induced by 70 mM KCl [21]. It was reversible and returned to basal levels after the withdrawal of αKG and was suppressed within 47% by 1 mM gabaculine. In parallel experiments in incubated and permeabilized islets, the ATP content and ATP/ADP ratio of islets, as well as the amount of ATP diffused and accumulated in the extracellular medium, were measured [21]. The islet ATP content was decreased by 1 mM gabaculine (*p* < 0.05), and the amount of the medium ATP was severely reduced by 37% (*p* < 0.0005). Neither the islet ATP content nor the amount of the medium ATP due to the metabolism of other tested Krebs cycle intermediary metabolites were significantly modified by gabaculine. The use of 10 mM SSA alone, which was surprisingly permeable through the plasma membrane, stimulated a biphasic secretion of insulin of lower magnitude than 20 mM glucose, and its second phase was partially suppressed by 1 mM gabaculine [14]. It also significantly increased the islet ATP content (+93.5 %) and the ATP/ADP ratio (+84%). At 1 mM glucose, 10 mM SSA depolarized the membrane potential of isolated β-cells and diminished membrane currents through K_ATP_ channels. The depolarization capacity of glucose was not altered after pre-incubating β-cells for 1–2 h with 1 mM gabaculine (14).

### 1.9. Conclusions

The GABA shunt in islet β-cells seems to contribute to the metabolic energy required for a sustained stimulation of insulin secretion by nutrient and metabolic secretagogues. As judged by the documented dependence of the metabolic oxidation of αKG and its ATP production on the functioning of the GABA shunt, its contribution might be significant.

### 1.10. Future Directions: Integral View of the Stimulus-Secretion Coupling Mechanism of Insulin Release

There seems to be a consensus on the metabolic nature of the amplifying phase of insulin secretion: an export of mitochondrial intermediary metabolites of the citric acid cycle to the cytosol (cataplerosis) would impact on a cytosolic target to maintain a sustained stimulation of the second phase of insulin secretion (L-glutamate, αKG, short-chain acyl-coA, and NDPH) [22]. Several candidates have been proposed for this role, but no one has yet been experimentally demonstrated to participate directly in the process of insulin secretion. Maintaining cataplerosis requires “anaplerotic reactions” that synthesize citric acid cycle intermediary metabolites to maintain the components of the cycle stable. In the case of glucose, the carboxylation of pyruvate to oxalacetate by pyruvate carboxylase (PC) is considered the main mechanism generating the required anaplerosis. It is estimated that more than 50% of glycolytic pyruvate is carboxylated by PC, whereas the other half is decarboxylated to acetyl-CoA by pyruvate dehydrogenase (Pdh) and oxidized in the citric acid cycle [23].

Attempts to check the functionality of PC-driven anaplerosis have generated contradictory results. The aim was to down-regulate PC gene expression through the neutralization of PC-mRNA with specific sirNAs and looking for the functional consequences on glucose-induced insulin secretion and islet metabolism. In one of the studies, blocking PC expression did not affect insulin secretion at all but increased it slightly but significantly [24]. In another study, the same maneuver resulted in a decrease in glucose-induced insulin secretion [25]. However, both studies coincided in the demonstration of an important increase in the lactic acid concentration. A further investigation is required for the quantification of PC gene expression, in comparison to Pdh, as well as their enzymatic activities and regulation. Moreover, it cannot be discarded that PC activity can be inhibited intracellularly by L-glutamate that behaves as a non-competitive inhibitor with respect to acetyl-CoA in vertebrates with a K_i_ around 4 mM [26].

As already commented in the Introduction, extracellular ATP reproduces a biphasic insulin secretion in KCl-permeabilized islets [5] and this argues against the possibility that a metabolite candidate would be required to potentiate the amplifying phase of insulin exocytyosis. However, more research is needed to corroborate which is the best candidate.

Notwithstanding, the uncovering of a new mitochondrial anaplerotic entrance of succinic acid in the citric acid cycle through the GABA shunt gives more alternatives to understanding how the β-cell “evaluates” the plasma glucose concentration. An exclusive or predominant use of the GABA shunt-dependent anaplerosis would reduce the accumulation and derivation of glycolytic pyruvate into anabolic metabolic pathways. This would reduce the subtraction of ATP for anabolic processes. It is known that gluconeogenesis is probably unsignificant in β-cells that do not express the phosphoenolpyruvate kinase enzyme (PEPCK) [27]. Β-cells are synthesizing phospholipids and triacylglycerols, as demonstrated by the incorporation of [U-^14^C] FFA and [U-^14^C] glucose, and this process is promoted by high glucose [28,29]. It might be discussed which of two possible processes is predominant, lipogenesis or esterification of FFA. Islet FFA oxidation is about 30-fold lower than glucose oxidation [30]. However, its quantitative importance has not been determined. Deviation of citric acid cycle intermediaries for the provision of non-essential aminoacids is possibly not strictly required, as the constant supply of plasma L-glutamine provides L-glutamate and L-aspartate.

The GABA shunt will theoretically exchange 1 mol of αKG against 1 mol of GABA and will not lead to an excessive accumulation of citric acid cycle intermediates. This would allow the β-cell to “sense” the flux of aerobic glycolysis with minimal interference of any other pathway. It is tempting to speculate that the β-cell evaluates the plasma glucose concentrations in proportion to the ATP generated by the metabolism of glucose in aerobic glycolysis.

## 2. Possible Implication of the GABA Shunt in β-Cell Degradation in Type 2 Diabetes

### 2.1. Introduction

Type 1 diabetes is an autoimmune disease caused by the activation of CD8+ and CD4+ T cells targeted to a group of autoantigens released from β-cells: (Pre)proinsulin, glutamic acid decarboxylase of 65 KD (GAD65), tyrosine phosphatase IA2, and the zinc transporter ZnT8. GAD 65 is one of the most prevalent autoantigens found in patients with type 1 diabetes [31].

### 2.2. Intracellular vs. Extracellular Effects of GABA

GABA is released in a pulsatile manner by non-diabetic human islets, and it is not modified by a range of glucose concentrations (1, 3, and 11 mM), as “detected by cytosolic Ca^2+^ flux in GABAB receptor-expressing biosensor cells or on total GABA secretion measured by HPLC” [8]. The lack of sensitivity of GABA release to glucose makes it difficult to understand how it might be synchronized with glucose-induced insulin secretion. In one study, inhibition of GABA biosynthesis with allylglycine decreased GABA release and stimulated basal serotonin/insulin secretion that “failed to display regular secretory pulses” [8]. By contrast, blocking islet GABA metabolism with 10 µM γ-vinyl-GABA (GABAT inhibitor) increased GABA release and decreased basal serotonin/insulin secretion in the presence of 3 mM glucose [8]. These results agree with the assumption that GABA release is governed by the intracellular content of the γ-amino acid. However, these inhibitors do not mimic the effect of insulin secretagogues that increase GABA metabolism and consequently decrease islet GABA content and its release, as commented on before. Otherwise, as islet GABA content depends on extracellular L-glutamine concentration, addition of a physiological (0.5–1.0 mM) or higher (10 mM) concentration of the α-amino acid would increase islet GABA content and, therefore, its release, resulting in the inhibition of high glucose-induced insulin secretion, which contrasts with accumulated experimental evidence. In fact, it might be considered that a physiological L-glutamine concentration should always be used to study the function of non-cultured islets in in vitro experiments. However, it cannot be denied that both intra- and extracellular functions of islet GABA might be concordant, mainly in the inhibitory side of the context.

Importantly, using immunostaining, islets from type 1 and 2 diabetic patient donors were shown to be depleted of GABA in β-cells despite the presence of GAD65 [8]. No pulsatile GABA secretion could be observed in type 2 diabetic islets, and it could be rescued by 10 µM γ-vinyl-GABA (a GABAT inhibitor). However, the periodicity of serotonin/insulin release was diminished (<0.01) by 10 µM γ-vinyl-GABA [8]. It might be interesting to examine whether there is any alteration in the gene expression or activity of GABA shunt enzymes that might contribute to the derangement of diabetic islets’ function.

### 2.3. Metabolic Changes Contributing to Islet Degradation in Type 2 Diabetes

In a recent comprehensive publication, the metabolic mechanism(s) that might be implicated in the degradation of the β-cells of type 2 diabetic islets due to persistent hyperglycemia were studied [32]. The authors found an elevated expression of most of the genes codifying glycolytic enzymes and a very strong suppression of glyceraldehyde phosphate dehydrogenase (GAPDH) activity. Hyperglycemia-induced consequences were prevented by D-mannoheptulose. The authors proposed the hypothesis that an increase in a glycolytic metabolite between glucokinase and GAPDH mediates the effects of hyperglycemia through the inhibition of AMPK and the activation of mTORC1 activities, respectively. Most of the consequences of hyperglycemia could be prevented by an inhibitor of one of the effectors activated by mTORC1, ribosomal protein S6 kinase (70S6K-Pi), which promotes the translation of mRNAs exhibiting specific sequences in their 5′ terminus. Besides glycolysis, hyperglycemia suppressed the expression of some of the genes encoding Krebs cycle enzymes and the expression of mitochondrial electron chain transporters [32], leading to the suppression of ATP-linked respiration. However, this downregulation of mitochondrial gene expression could not be prevented by S6K inhibitors, such as in the case of glycolysis. It is unknown whether GAPDH enzymatic activity may be restored by S6K inhibitors in diabetic islets or INS-1 cells cultured at 25 mM glucose for 48 h.

Islets and INS-1 cells are characterized by a “reverse” Pasteur effect when subjected to anoxia (under N_2_): their glucose utilization rate at high glucose levels is almost completely depressed instead of being stimulated [33]. This “anomalous” feature of β-cells has been attributed to their low lactic acid dehydrogenase activity which cannot cope with the re-oxidation of cytosolic NADH at high rates of glycolysis [34]. This function is taken over by the glycerol phosphate shuttle coupled to the respiratory chain. It might be concluded that most of the islet glucose utilization rate is performed as aerobic glycolysis that depends on the integrity of mitochondrial respiration. This suggests that a mitochondrial respiratory defect might be the primary cause of islet degradation in diabetic islets. This might lead to a block of GAPDH and of the reoxidation of glycolytically produced NADH, followed by an upregulation of the gene expression of some glycolytic genes and the already described consequences [32].

### 2.4. Conclusions and Perspectives

In our opinion, there is no consensus regarding the priority of an autoimmune attack against GAD65 vs. a pre-existing metabolic derangement of β-cells in the development of type 1 diabetes. Studies involving patients with type 1 diabetes suggest that “a 2-component causal model for T1D comprising constitutional metabolic impairments that act in concert with autoimmunity” might be responsible for the development of this disorder [30]. Moreover, studies in non-inbred BB rats (BB/Hagedorn, a model of spontaneous autoimmune type 1 diabetes) suggest that “beta cells may have an inherent sensitivity that possibly makes them susceptible to autoimmune attack” [35]. In contrast, in type 2 diabetic patients, several risk factors have been identified that might primarily provoke a functional derangement of the β-cells [30].

In conclusion, many experimental data support the role of the GABA shunt metabolism in the stimulation of insulin secretion. The fact that GAD65 is one of the most prevalent autoantigens in type 1 diabetic patients, which is immunologically destroyed in the development of this disorder, may indicate that the blocking of the GABA shunt might be co-responsible for β-cell degradation. As the GABA metabolism, together with that of glucose, might contribute to maintaining mitochondrial ATP production, its failure might provoke anoxic-like conditions in beta-cells; despite a normal supply of oxygen, it would not be optimally utilized because of the diminished oxidative phosphorylation (decreased mitochondrial respiration). Therefore, any enzyme of the GABA shunt might be considered as a risk factor for the triggering of β-cell malfunction, including GAD65, GABAT, SSA-dehydrogenase (NADH-dependent, generating succinic acid) and, perhaps, SSA reductase (NADP^+^-dependent, producing γ-hydroxybutyric acid (GHB)). The latter enzyme was surprisingly strongly inhibited (−98%) by 10 mM KIC without affecting its competitor enzyme (SSA dehydrogenase), which competes for their common substrate SSA [14].

## Abbreviations

gamma-aminobutyric acid (GABA); semialdehyde succinic acid (SSA); succinic acid (SUCC); α-ketoisocaproic acid (KIC); α-ketocaproic acid (KC); 2-oxoglutarate or α-ketoglutarate (2-OG or α-KG); gamma-hydroxybutyric acid (GHB); 2-OG-dehydogenase (2-OG-dh); SSA dehydrogenase (SSA-dh); SSA reductase (SSA-red); GABA transaminase (GABAT); glutamate dehydrogenase (GDH); 4-methyl valeric acid (MVA).
